# Supplementary material for: Function, structure and quality of striated muscles in the lower extremities in patients with late onset Pompe Disease—an MRI study
Source: PeerJ. 2021 May 6;9:e10928. doi: 10.7717/peerj.10928 (PMC8106912; doi:10.7717/peerj.10928)
Supplement: Supplemental Information 1 [file peerj-09-10928-s001.docx]

Protocol:

**Function, structure and quality of striated muscles in patients with**

**Muscular diseases - an MRI study on Pompe Disease and Dystrophia Myotonica**

Project manager,

Contact: Rosa Andersen Mencagli

Department of Neurology, Aarhus University Hospital

Principal supervisor:

Professor, consultant, dr. med. Phd Henning Andersen

Department of Neurology, Aarhus University Hospital

Co-supervisor:

PhD-student, MSc Michael Væggemose,

Department of Neurology, Aarhus University Hospital

**Aim**

To evaluate muscle quality in patients with late onset Pompe disease (PD) in comparison to healthy controls by determining muscle strength in relation to muscle size.

Furthermore, to evaluate muscle quality in patients with late onset PD before and following enzyme replacement therapy (ERT).

To evaluate muscle quality in patients with Dystrophia Myotonica in comparison to healthy controls by determining muscle strength in relation to muscle size.
Compare the findings from the two patient groups.

**Introduction:**

**Pompe Disease**

Pompe Disease (PD), also known as Acid Maltase Deficiency (AMD) or Glycogen Storage Disease type II, is a rare autosomal recessive disorder, caused by lack of the lysosomal enzyme α-glucosidase (AAG). The enzyme is responsible for metabolizing lysosomal glycogen. The lack of the enzyme results in glycogen accumulation within the lysosomes, which ends in cellular damage and tissue damage^[[1]](#endnote-1)^. The estimated frequency of the disease is 1/40,000 in Netherlands^[[2]](#endnote-2)^, and similar or lower frequencies have been reported for other countries^[[3]](#endnote-3)^ ^[[4]](#endnote-4)^.

The disease presents in a wide range of phenotypes, differing in age at onset, severity of symptoms and disease progression. The most severe form is the infantile form, characterized by complete lack of AAG presenting at 1 to 2 months of age. It especially affects the heart and skeletal muscle and death occur before the age of 2 years due to cardiorespiratory failure.

The less severe form, where residual activity of AAG is still detectable, can manifest from young

age to adulthood (late onset Pompe Disease). Symptoms are generally related to skeletal muscle dysfunction, resulting in fatigue, muscle weakness and respiratory deficiency^[[5]](#endnote-5)^ ^[[6]](#endnote-6)^.

**Enzyme Replacement Therapy and disease monitoring**

In 2006 Enzyme Replacement Therapy (ERT) with recombinant AAG was approved for Infantile PD.

Several studies has since shown good therapeutic effect of the recombinant alglucosidase also for late onset PD^[[7]](#endnote-7)^ ^[[8]](#endnote-8)^ ^[[9]](#endnote-9)^.

The main endpoints have been the 6 minute walking test (6MWT) and vital capacity (VC). However, the studies underline the heterogeneity of clinical manifestation of disease and of ERT outcome, and that monitoring of disease progression and effect of ERT is needed.

A new emerging and promising tool for disease monitoring is magnetic resonance imaging (MRI). This technique has been useful in many neuromuscular disorders. The technique is non-invasive, has high spatial resolution and introduces no risk to the patients (especially ionizing radiation exposure). Since MRI has good soft-tissue contrast it is useful for identification of fat replacement in muscles^[[10]](#endnote-10)^ ^[[11]](#endnote-11)^.

**Magnetic Resonance Imaging and Pompe Disease**

In chronic diseases, muscle involvement detected by MRI is based principally on signal intensity changes resulting from fat infiltration into muscle combined with decreased muscle volume. It is therefore possible to observe muscle involvement of the disease, which overall follows a characteristic pattern^[[12]](#endnote-12)^.

In late onset PD Pichiecchio et al., found that the MRI score related to the severity of muscular abnormalities and to the patients’ loss of muscular strength (MRC scale)^[[13]](#endnote-13)^. The same correlation between muscle involvement on MRI and muscle strength was reported by Vielhaber et al.^[[14]](#endnote-14)^ and Alejaldre et al^[[15]](#endnote-15)^.

The research group of Pichiecchio et al. also used MRI to evaluate the effects from 6 months of ERT, and found that ERT results in increased muscle mass, subcutaneous fat and intramuscular fat. Quantitative increase in the muscle bulk was mainly observed in the spared muscle^[[16]](#endnote-16)^.

Moreover it seems that changes in muscle mass positively correlate to changes in muscular strength (measured on hand-held dynamometer), and that those changes can be detected after one year of ERT therapy^[[17]](#endnote-17)^.

A recent Danish study had different findings^[[18]](#endnote-18)^. Interestingly, weak muscles seemed to benefit more from ERT than less affected ones, which is in contrast to previous findings. Furthermore, flexor muscle groups had greater response to ERT, contrasting previous studies reporting greater effect of the extensor muscle groups.

These contradictory findings could be due to differences in design and methodology (for example in measuring muscle strength and choosing different sequences of muscle-MRI) or it could be due to the heterogeneity in the clinical presentation of PD.

Therefore, more studies are needed, in order to determine the relation between the effect of ERT on muscle mass and muscle strength in PD patients.

**Dystrophia Myotonica**

an autos o-

mal dominant multisystemic disorder, is the most

common adult for m of muscular dystrophy, af fect-

ing approximately 1 in 7400

an autos o-

mal dominant multisystemic disorder, is the most

common adult for m of muscular dystrophy, af fect-

ing approximately 1 in 7400

Dystrophia Myotonica is an autosomal dominant progressing multisystemic disease. It is the most common adult form of muscular dystrophy. In Denmark there are approximately 1500 people living with the disease^[[19]](#endnote-19)^.
As with Pompe disease, the muscular tissue slowly degenerate and fat infiltration occur.
As it is the case with other muscular diseases, there is an increasing interest for MR imaging as a useful non-invasive toll for monitoring the disease and for monitoring the outcomes of the increasing numbers of clinical trials on Dystrophia Myotonica^[[20]](#endnote-20)^.

**Hypothesis**

In this study, we want to test the following hypotheses:

- Intrinsic muscle strength in patients with Pompe disease and Dystrophia Myotonica is linear correlated with muscle to fat volume ratios from MR images.
- Disease grade in patients with Pompe disease and Dystrophia Myotonica corresponds to the extent of fat proliferation in the muscles of the lower extremities.
- Intrinsic muscle strength is a more specific and sensitive method to reveal the effects of enzyme replacement treatment in patients with Pompe than the six minutes-walk-test.

**Method**

**Study population**

Pompe disease population

Fifteen patients with PD and 15 healthy age matched control subjects will be included. All patients with PD in Denmark (9) will be invited to participate, irrespective of whether they are given ERT or not. The remaining participants will be recruited among PD patients from the northern part of Germany (the Hamburg and Schleswig- Holstein area). Patients will be evaluated at inclusion and again after 10 months.
In case of de novo patients an additional examination date will be performed following 6 months of treatment. This will provide detailed new results of muscle structure and function in the early stages of treatment, before and after initial ERT. This provides the opportunity to monitor the effects on ERT during the first year of treatment, which is the period where the greatest effect of ERT on muscle strength has been found.

In addition to the MRI and Dynamometer measurement, participants will be evaluated with a 6 min. walking test, vital capacity and negative inspiration/expiration test, sit-to-stand test as well as a structured neurological examination.

Dystrofia Myotonica population

Fifteen patients with Dystrophia Myotonica will be included. Subjects will be recruited via their attendance at the hospital clinic of Aarhus University Hospital.

INCLUSION CRITERIAS

- Diagnosed Pompe disease patients, whether they are in treatment with ERT or not.
- Diagnosed Dystrophia Myotonica patients.
- Healthy controls, have to be age-matched to patients with muscular disease.
- Age above 18

EXCLUSION CRITERIAS

- The subjects must be able to perform an MR-scanning. Moreover they have to sign and respect the MR security rules “Kontrol skema før MR undersøgelse” and "Patient information i forbindelse med MR scanning”
- The subjects must be capable of performing the muscle strength test by the Dynamometer.

**MR protocol**

The MR protocol includes the special T1 weighted sequence DIXON (1x1x3mm^3^; TR: 5.31ms TE: 2.46ms) enabling acquisition of muscle and fat images at 3 Tesla (Magnetom-Skyra, Siemens AG, Erlangen, Germany). The MR scans will consist of 400 axial slices covering from L1 to the ankle resulting in a total scan time of 22½ minutes.


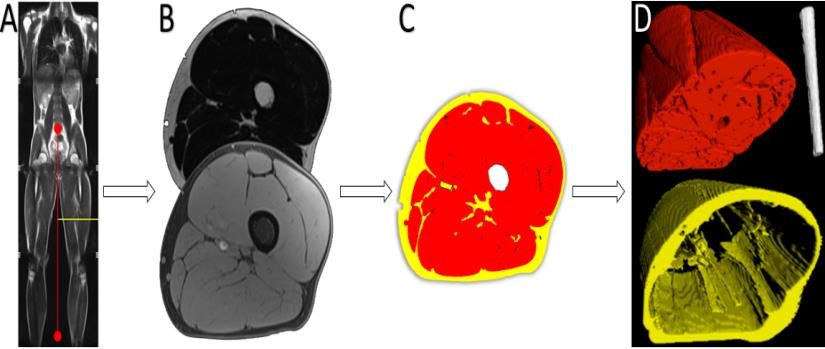


**FIGURE 1 – A) FAST VIEW WHOLE-BODY CORONAL SCAN, RED LINE INDICATES THE REGION-OF-INTEREST FROM L1 TO THE ANKLE, USED IN THE DIXON SCAN. B) DIXON SCAN OF THIGH IN HEALTHY SUBJECT. C) SEGMENTATION OF MUSCLE, FAT AND BONE MARROW. D) VOLUMETRIC MODELLING OF MUSCLE, FAT AND BONEMARROW (RESULTS SEEN IN TABLE 2)**

**Isokinetic Dynamometry**

Muscle strength will be measured by isokinetic dynamometry. The measurement will be done using the Biodex System 3 PRO Dynamometer (Biodex Medical Systems Inc. NY, USA) for which a standardized protocol has been developed.

Maximal isokinetic strength of extensors and flexors of the dominating arm and extensor and flexors at the knee, ankle and hip of the non-dominant lower extremity will be measured.

**Risk, side effects and disadvantages**

There are no known side effects related to MR-scanning.
During the examination, the subjects will wear headphones in order to reduce the high sounds that comes from the scanning machine.
The scan is painless and there is no use of contrast. The only disadvantage is that the person has to lie still for 20-30 minutes.
There are some security rules to respect in order to avoid accidents due to metals being attracted by the machine’s strong magnetic field.

No side effects are associated to muscle strength measurement with the Dynamometer.
However, weak patients may be a bit tired after the examination, as they have to perform some movements using all their force.

**Statistics**

Comparison between baseline and visits will be performed using conventional statistic methods as unpaired t-test or non-parametric tests such as Wilcoxon signed-rank test. In variation analyses of several groups ANOVA and ANCOVA will be used. A significant difference of p-values below 0.05 has to be determined, to determine the validity of the findings. With a significant level of 5% a power calculation estimated a needed sample size of 14 patients, with a power of 90%. The calculation is based on an expected decrease in muscle volume compared to fat (1.2 vs 0.95 with a standard deviation of 0.2). The power calculation is an estimate, but compared to a similar study^xiii^ the sample size includes an extra 3 subjects. Given the rare condition of PD, inclusion of patients will continue even if the required minimum is achieved.

**Timetable**

The research year will begin 1-09-2015 and will end 31-08-2016.

**Feasibility**

Patients will be recruited from the Departments of Neurology at Aarhus University Hospital, Rigshospitalet, and Aalborg University Hospital. The main investigator is currently at her 9. semester in medicine at Aarhus University. The study will be performed at the Department of Neurology and MR Centre at Aarhus University Hospital.

The MR scanning will take place at Skejby University Hospital. The research year student, Rosa Andersen Mencagli, will perform the scans, under the supervision of PhD student Michael Væggemose (co-supervisor). At the department of Neurology in Aarhus there is a Biodex isokinetic dynamometer.

**Dissemination and publications**

The results will be published in international journals under the Vancouver rules regardless of whether the results are positive, negative or inconclusive. Results of the study will form the basis for a research year thesis, with an oral presentation as examination.

**Ethical consideration**

The project will be approved by national guidelines and by *Datatilsynet*.
Personal information will be treated confidentially, according to the law.
The project is not connected to any ethical issues. There are no side effects related to the examinations.
Moreover, for patients with Pompe disease, the examinations are part of the treatment and monitoring offer, from the national health system. Participating in the projects will simply mean closer contact to the health system for one year, which can be a benefit for the patient, since possible new complications can be discovered, investigated and treated early on.
Due to the rareness of Pompe disease it is difficult to make studies with sufficient number of participants. Therefore, participating in the project also has a direct impact on patients suffering from Pompe disease, as new knowledge about the disease is fundamental for better understanding of the disease and treatment.
Healthy control persons have the opportunity to get an MR-imaging of their body (from L1 down to the feet). In case of any abnormal findings, the subjects will have the possibility of early treatment by referral to their physician.

**Perspective**

The study will contribute to a more sensitive diagnosis of PD using specialized MR imaging for future clinical practice. The project will furthermore produce a better clinical understanding of this rare complication and provide the basis for data to evaluation of the disease progression.

Moreover, if the study shows there to be a correlation between muscle strength and MR imaging of muscle/fat ratio in patients with PD and Dystrophia Myotonica, the technique could also be used for monitoring other patient groups with similar myopathic disorders.

**Budget**

Professor Dr. med Henning Andersen is the initiator of the project, together with Phd, MSh Michael Væggemose.

The project is seeking funds for 12 months of salary for medical student Rosa Mencagli Andersen for a total of 120.000 DKK.

**Remuneration**

Expences related to the project will be covered by the departments involved.
The researchers will not take any economical advantage from the study.
Expenses for transport related to the examinations, will be refunded according to the department’s guidelines.
Compensation for participating in the project will be given according to national guidelines, and has to be reported to SKAT as B-income.

**Informed consent**

Subjects will be recruited on private initiative.
Potential subjects with Pompe Disease and Dystrophia Myotonica will be identified through their attendance at the neurological clinic. They will receive written information about the project alongside the answering papers and the request to contact the project manager, Rosa Andersen Mencagli, by mail, e-mail or telephone, if more information is desired.
Healthy control persons will be recruited through notices on public bulletin boards such as *Aarhus Onsdag* and internal pages on *forsøgsperson.dk* and *sunhed.dk.* Interested persons will be encouraged to contact the project manager by e-mail or telephone to hear more about the project. Afterwards, if they still are interested, they will receive more written information about the project, together with VEK´s appendix: “forsøgspersonens rettigheder i et sundhedsvidenskabeligt forskningsprojekt”, as well as the proposal for the informative conversation. The proposal will tell potential subjects about the right to have a period of reflection after the informational conversation, and that they can bring a companion to that informative conversation.

The informative conversation will be conducted by the research year student, Rosa Andersen Mencagli, who via her close connection to the study, and knowledge of the field, has the necessary professional qualifications. The conversation will take place in a meeting room at the Aarhus University Hospital. The potential subjects will be reminded that it is an informative conversation. Information about the project will be given in an easy every-day language, without the use of technical words or value loaded sentences. There will be time enough for a detailed explanation and for questions. The conversation will be adjusted to the recipients conditions, such as experience, age etc. The conversation will be based on the written informational papers, and will include information about possible predictable risks, side effects, complications and disadvantages related to participation in the projects, as there could emerge unpredictable risk and complications related to the project. Moreover, the potential subjects will be informed that their personal information can be passed on to, and processed by the people responsible of the quality control of the project, which is compulsory by law. The potential subjects will also be informed of the possibility to decline knowledge about new healthy issues during the project.

After the informative conversation the potential subjects have at least one week of reflection, before signing the informed consent. The subjects have the possibility to withdraw from the project at any time without any consequences for them.

Information from the patient records is desired, as has been explained in the informational papers.
In the case that new knowledge about effects, risks, complications and disadvantages arise during the project, the subjects will be informed. They will also be informed if the project design changes and the change has consequences for the subject´s security. If new findings concerning the health of the subject occur during the project, the subject will be informed, unless he/she has previously expressed the wish to not be informed about new medical issues.
The subjects will be informed about the project’s results together with possible individual consequences. At the end of the project, a short and easy understandable report will be written in Danish, summarizing the project’s findings. The subjects can get a copy of the report by writing to the contact person of the project (Rosa Andersen Mencagli). If the project for any reason should be discontinued, the subjects will be informed about it.

**References:**

1. Hirschhorn R, Reuser A J J. Glycogen Storage Disease Type II (GSDII). Scriver CR, Beaudet AL, Sly WS, Valle D, eds. The Metabolic and
   Molecular Bases of Inherited Disease. 8th edn. NY: McGraw-Hill, 2001: 3389–420. [↑](#endnote-ref-1)
2. Ausems MG, Verbiest J, Hermans MP, et al. Frequency g glycogen storage disease type II in the Netherlands:implications for diagnosis and genetic counselling. Europ. J. Hum Genet. 1999; 7:713-716. [↑](#endnote-ref-2)
3. Martiniuk F, Chen A, Mack A et al. Carrier frequency for glycogen storage disease type II in New York and estimates of affected individuals born with the disease. Am J Med Genet 1998; 79: 69-72. [↑](#endnote-ref-3)
4. Meikle P J, Hopwood J J, Clague A E, Carey W F. Prevalence of lysosomal storage disorders. JAMA 1999; 281; 249-254. [↑](#endnote-ref-4)
5. Van der Ploeg A T, Reuser A J J. Pompe’s disease. (2008) Lancet 372:1342-53. [↑](#endnote-ref-5)
6. Hagemans M L, Winkel L P, Doorn P A, et al. Clinical manifestation and natural course of late-onset Pompe's disease in 54 Dutch patients. Brain 2005; 128:671–677. [↑](#endnote-ref-6)
7. Van der Ploeg A T, Clemens P R, Corzo D. et al. A randomized study of alglucosidase alfa in late-onset Pompe’s disease. N Engl J Med 2010; 362(15):1396–1406. [↑](#endnote-ref-7)
8. Toscano A, Schoser B. Enzyme replacement therapy in late-onset Pompe disease: a systematic literature review. J Neur6ol.2012; 20(4):951– 959. [↑](#endnote-ref-8)
9. Anderson L J, Henley W, Wyatt K M, et al. Effectiveness of enzyme replacement therapy in adults with late-onset Pompe disease: results from the NCS-LSD cohort study. J Inherit. Metab. Dis. 2014; 37:945–952. [↑](#endnote-ref-9)
10. Mercuri E, Pichiecchio A, Allsop J, Messina S, Pane M, Muntoni F. Muscle MRI in inherited neuromuscular disorders: past, present, and future. J Magn. Reson. Imaging 2007; 25:433–40. [↑](#endnote-ref-10)
11. Ponrartana S, Ramos-Platt L, Wren T A, Hu H H, Perkins T G, Chia J M, Gilsanz V. Effectiveness of diffusion tensor imaging in assessing disease severity in Duchenne muscular dystrophy: preliminary study. Pediatr. Radiol. 2014; DOI 10.1007/s00247-014-3187-6. [↑](#endnote-ref-11)
12. Carlier R Y, Laforet P, Wary C, Mompoint D, Laloui K, Pellegrini N, Annane D, Carlier P G, Orlikowski D. Whole-body muscle MRI in 20 patients suffering from late onset Pompe disease: Involvement patterns. Neuromuscul. Disord. 2011; 21:791-9. [↑](#endnote-ref-12)
13. Pichiecchio A, Uggetti C, Ravaglia S, Egitto M G, Rossi M, Sandrini G, et al. Muscle MRI in adult-onset acid maltase deficiency. Neuromuscul. Disord. 2004; 14:51–55. [↑](#endnote-ref-13)
14. Vielhabera S, Brejovaa A, Debska-Vielhabera G, Kaufmanna J, Feistnera H, Schoenfelda M A, Awiszus F. 24-Months results in two adults with Pompe disease on enzyme replacement therapy. Clinical Neurology and Neurosurgery 2011; 113 350–357 Contents. [↑](#endnote-ref-14)
15. Alejaldre A, Díaz-Manera J, Ravaglia S, et al. Trunk muscle involvement in late-onset Pompe disease: study of thirty patients. Neuromuscul Disord. 2012; 22(Suppl 2):S148–S154. [↑](#endnote-ref-15)
16. Pichiecchio A, Poloni G U, Ravaglia S. Enzyme replacement therapy in adult-onset glycogenosis II: is quantitative muscle MRI helpful? Muscle Nerve 2009; 40:122–5. [↑](#endnote-ref-16)
17. Ravaglia S, Pichiecchio A, Ponzio M, Danesino C, Saeidi Garaghani K, Poloni G U, Toscano A, Moglia A, Carlucci A, Bini P, Ceroni M, Bastianello S. (2010) Changes in skeletal muscle qualities during enzyme replacement therapy in late- onset type II glycogenosis: temporal and spatial pattern of mass vs. strength response, J. Inherit. Metab. Dis. 33 737–745. [↑](#endnote-ref-17)
18. Andreassen C S, Schlütter J M, Vissing J, Andersen H. (2014) Effect of enzyme replacement therapy on isokinetic strength for all major muscle groups in four patients with Pompe disease-a long-term follow-up. Mol. Genet. Metab. 112(1): 40-3. [↑](#endnote-ref-18)
19. https://www.sundhed.dk/sundhedsfaglig/laegehaandbogen/neurologi/tilstande-og-sygdomme/muskelskelet/muskelsygdomme-arvelige/#10. [↑](#endnote-ref-19)
20. B Hiba, N Richard, L J. Hébert, C Coté, M Nejjari, C Vial, F Bouhour, J Puymirat, M Janier. Quantitative assessment of skeletal muscle degeneration in patients with myotonic dystrophy type 1 using MRI. J. Magn. Reson. Imaging 2012;35:678-685. © 2011 Wiley Periodicals, Inc. [↑](#endnote-ref-20)
